# Phase I study of alpha-tocopherlyoxyacetic acid in patients with advanced cancer

**DOI:** 10.1186/2051-1426-3-S2-P148

**Published:** 2015-11-04

**Authors:** Brendan D Curti, Emmanuel Akporiaye, Kim Sutcliffe, Keith S Bahjat, Yoshinobu Koguchi, Julie Cramer, Walter Urba

**Affiliations:** 1 Providence Cancer Center, Providence Portland Medical Center,, Portland, OR, USA; 2 Sidra Medical and Research Center, Doha, Qatar; 3 Providence Portland Medical Center, Portland, OR, USA; 4 Earle A. Chiles Research Institute, Robert W. Franz Cancer Research Center, Providence Cancer Center, Portland, OR, USA; 5 Earle A. Chiles Research Institute, Portland, OR, USA

## 

Alpha-tocopheryloxyacetic acid (α-TEA) is a pro-apoptotic agent that demonstrates in vivo anti-tumor activity in pre-clinical models in a T cell-dependent fashion. In IND-enabling studies, orally administered α-TEA lysine salt demonstrated a safety profile in mice and dogs providing rationale for initiating a Phase I clinical trial of α-TEA in patients with advanced cancer (NCT02192346). α-TEA lysine salt was administered to patients in gelatin capsules daily for 28 days. The main clinical objectives of the trial are to characterize α-TEA related toxicity, and determine the maximum tolerated dose, and pharmacokinetics of a-TEA in humans. Secondary objectives are to characterize the phenotype of T-cell subsets in the peripheral blood by flow cytometry. The diagnoses of patients in the ongoing trial include renal cancer, esophageal cancer, thyroid cancer, duodenal cancer, squamous cell oropharyngeal cancer and advanced squamous cell carcinoma of the head and neck. Six patients have been treated at the 2.4mg/kg dose level, and five patients at the 4.8mg/kg dose level. Two patients had atrial fibrillation possibly associated with α-TEA at the 4.8 mg/kg dose level. Grade 2/3 toxicities (24 hour diarrhea) were observed in Subject 1 that in retrospect was not α-TEA related. Subject 3 had grade 4 elevations of hepatocellular enzymes on day 28 from disease progression and grade 3 transient lymphopenia, possibly related to α-TEA. Three patients have stable disease, one lasting 5 months and another lasting 2+ months, which is ongoing. Of interest is that the two patients with stable disease demonstrated an expansion of the CD8^+^ T cell subset and another patient demonstrated increased CD8 effector memory and substantial increase in expression of the activation markers, CD38 and HLA-DR at day 8 post-treatment. The trial is still accruing patients and the pharmacokinetics, pharmacodynamics, and identification and characterization of α-TEA metabolites in human plasma are ongoing.

## Trial registration

ClinicalTrials.gov identifier NCT02192346.

